# *miR-550a-3p* is a prognostic biomarker and exerts tumor-suppressive functions by targeting *HSP90AA1* in diffuse malignant peritoneal mesothelioma

**DOI:** 10.1038/s41417-022-00460-7

**Published:** 2022-03-29

**Authors:** Rihan El Bezawy, Stefano Percio, Chiara Maura Ciniselli, Michelandrea De Cesare, Gennaro Colella, Matteo Dugo, Silvia Veneroni, Valentina Doldi, Silvia Martini, Dario Baratti, Shigeki Kusamura, Paolo Verderio, Marcello Deraco, Paolo Gandellini, Nadia Zaffaroni, Valentina Zuco

**Affiliations:** 1 Molecular Pharmacology Unit, Fondazione IRCSS Istituto Nazionale dei Tumori, 20133 Milan, Italy; 2 Bioinformatics and Biostatistics Unit, Fondazione IRCSS Istituto Nazionale dei Tumori, 20133 Milan, Italy; 3 Department of Medical Oncology, IRCCS Ospedale San Raffaele, 20132 Milan, Italy; 4 Biomarkers Unit, Fondazione IRCSS Istituto Nazionale dei Tumori, 20133 Milan, Italy; 5 Peritoneal Surface Malignancies Unit, Fondazione IRCSS Istituto Nazionale dei Tumori, 20133 Milan, Italy; 6 Department of Biosciences, University of Milan, 20133 Milan, Italy

**Keywords:** Cancer genetics, Mesothelioma

## Abstract

Diffuse malignant peritoneal mesothelioma (DMPM) is a rare and rapidly lethal tumor, poorly responsive to conventional treatments. In this regards, the identification of molecular alterations underlying DMPM onset and progression might be exploited to develop novel therapeutic strategies. Here, we focused on *miR-550a-3p*, which we found downregulated in 45 DMPM clinical samples compared to normal tissues and whose expression levels were associated with patient outcome. Through a gain-of-function approach using miRNA mimics in 3 DMPM cell lines, we demonstrated the tumor-suppressive role of *miR-550a-3p*. Specifically, miRNA ectopic expression impaired cell proliferation and invasiveness, enhanced the apoptotic response, and reduced the growth of DMPM xenografts in mice. Antiproliferative and proapoptotic effects were also observed in prostate and ovarian cancer cell lines following *miR-550a-3p* ectopic expression. *miR-550a-3p* effects were mediated, at least in part, by the direct inhibition of *HSP90AA1* and the consequent downregulation of its target proteins, the levels of which were rescued upon disruption of miRNA-*HSP90AA1* mRNA pairing, partially abrogating *miR-550a-3p*-induced cellular effects. Our results show that *miR-550a-3p* reconstitution affects several tumor traits, thus suggesting this approach as a potential novel therapeutic strategy for DMPM.

## Introduction

Diffuse malignant peritoneal mesothelioma (DMPM) is a rare tumor that develops from the mesothelial cells lining the peritoneal cavity. DMPM includes three histological subtypes, epithelioid, sarcomatoid, and biphasic (epithelioid and sarcomatoid), with the epitheliod subtype being the most frequent and less aggressive subtype [1]. Although locally aggressive, DMPM is characterized by poor prognosis, and patient survival does not exceed 1 year following treatment with palliative surgery and systemic or intraperitoneal chemotherapy [2]. The only treatment that meaningfully impacts the natural history of DMPM is aggressive cytoreductive surgery (CRS) combined with hyperthermic intraperitoneal chemotherapy (HIPEC), which is currently regarded as the gold-standard initial treatment for selected DMPM patients as it was found to significantly extend median survival time to 34–92 months [3–6]. However, for recurrent patients and for those who are not eligible for CRS + HIPEC there is an urgent need for alternative effective treatments.

DMPM is an understudied disease and, although there might be an association with asbestos exposure, its pathogenesis is mostly unknown. A better understanding of the disease biology, leading to the identification of molecular alterations underlying disease onset and progression, could provide a source of novel therapeutic targets. A few studies carried out thus far indicated that a fraction of DMPM is characterized by the presence of mutations in *BRCA1 associated protein 1*,*Neurofibromin 2*, *DEAD-Box Helicase 3 X-Linked*, *SET Domain Containing 2,* and *Histone Lysine Methyltransferase* genes, as well as by the loss of 3p21 locus, which includes chromatin modifiers and epigenetic regulatory genes [7, 8]. In addition, *ALK* rearrangements have been described in a small subset of younger women affected by DMPM [9]. However, it is still unclear whether such molecular alterations are causative of the disease or impact disease progression.

MicroRNAs (miRNAs) are small non-coding evolutionarily conserved RNA molecules, involved in post-transcriptional gene silencing by binding to 3’untranslated region (3’UTR) of target mRNAs, thus controlling a variety of important biological processes [10, 11]. Dysregulated miRNAs have been causatively associated with the pathogenesis of several diseases, including cancer. Depending on their expression levels, cellular context, and target genes, miRNAs can act as oncogenes or tumor suppressors [12]. In recent years, miRNA functional involvement in human cancer has raised an increasing interest toward their exploitation as therapeutic targets and tools [13]. Thus far, very little information is available on the expression and functional role of miRNAs in DMPM. Indeed, the current knowledge is limited to two miRNAs, *miR-34a* and *miR-380-5p*, that are negligibly expressed in DMPM and the ectopic expression of which in DMPM cell models exerts tumor-suppressive effects dealing with the inhibition of *MET* and *AXL* expression [14] and the perturbation of telomerase activity [15], respectively.

In the current study, we investigated the expression levels of *miR-550a-3p* and its association with clinical outcome in a cohort of DMPM patients who underwent CRS + HIPEC. Furthermore, we assessed the biological effects induced by *miR-550a-3p* ectopic expression in DMPM patient-derived cell lines to provide the preclinical basis for the design of a novel miRNA-based therapeutic approach. In addition, to broaden the relevance of our findings, we extended the analysis of *miR-550a-3p*-induced effects to cell lines of other human tumor types, such as ovarian and prostate cancer. Results are reported herein.

## Materials and methods

### Study population

Frozen DMPM lesions from 45 adult patients treated with CRS + HIPEC from 1997 to 2013 at the Fondazione IRCCS Istituto Nazionale dei Tumori (INT) of Milan were available for *miR-550a-3p* expression analysis. The H&E stained slides of all cases were reviewed, and the tumors were classified as epithelial, sarcomatoid, or biphasic according to the WHO classification. Eleven normal peritoneum specimens were also obtained from patients who underwent surgery for non-oncologic diseases. The study was approved by the Institutional Review Board. Written informed consent was obtained from all patients to donate the leftover tissue to INT after diagnostic and clinical procedures.

### Cell culture and transfection procedures

The DMPM cell lines STO, MP8, and MP115 were established in our laboratory from clinical samples of epithelioid (STO and MP8) and biphasic (MP115) DMPM [14–18]. Human prostate carcinoma cell lines, DU145 and PC3, were purchased from the American Type Culture Collection (ATCC, Manassas, VA, USA). A human ovarian carcinoma cell line, IGROV-1, was established from a patient with ovarian adenocarcinoma as described [19], and its resistant subline IGROV-1/Pt1 was generated by continuous exposure of parental cells to platinum drugs and was characterized by mutations in the *TP53* gene [19, 20]. DMPM cells were cultured in DMEM F-12 medium (Lonza, Basel, Switzerland). Ovarian and prostate cells were maintained in RPMI 1640 medium (Lonza). Both media were supplemented with 10% fetal bovine serum in a 37 °C humidified 5% CO_2_ incubator. All cells were human mycoplasma-free. All cell lines were authenticated by single tandem repeat analysis by the AmpFISTR Identifiler PCR amplification kit (Applied Biosystems, Waltham, Massachusetts, USA).

Synthetic *miR-550a-3p* mimic (hereafter *miR-550a-3p*) and mimic negative control (Neg) were purchased as Pre-miR™ miRNA precursor molecules (Thermo Fisher Scientific Inc, Waltham, Massachusetts, USA). Cells were transfected for 24 h with 20 nM of *miR-550a-3p* or miR-Neg, using Lipofectamine® RNAiMAX Transfection Reagent (Thermo Fisher Scientific Inc) with Opti-MEM I (Gibco, NY, USA) according to the manufacturer’s instructions.

In miR-Mask experiments, 20 nM of *HSP90AA1*-miScript Target Protector (Qiagen, Hilden, Germany) was transfected alone or in combination with *miR-550a-3p* mimic, under the same transfection conditions described above.

### RNA Extraction and qRT-PCR

Total RNA from tissue specimens and cell lines was isolated using the miRNeasy Mini Kit (Qiagen) according to the manufacturer’s guidelines, and 1 μg of total RNA was reverse transcribed to cDNA by miScript II RT Kit (Qiagen). *miR-550a-3p* and *HSP90AA1* mRNA expression levels were quantified by quantitative RT-PCR (qRT-PCR) using miScript SYBR Green PCR Kit (Qiagen) and TaqMan®gene expression assays (Thermo Fisher Scientific Inc), respectively (detailed in Material and Methods Supplementary Information). The primers for qRT-PCR were as follows: miScript Primer Assays specific for *miR-550a-3p* (MS00023807) and normalized on *SNORD48* (MS00007511) (Qiagen); *HSP90AA1* (Hs00743767, Thermo Fisher Scientific Inc) and normalized on *GAPDH* (Hs.PT.39a.22214836, Integrated DNA Technologies, Inc. Coralville, Iowa, USA). Amplifications were run on the 7900HT Fast Real Time PCR System (Applied Biosystem). Data were analyzed by SDS 2.2.2 software (Thermo Fisher Scientific Inc) and reported as 2^−ΔCt^ or as relative quantity (RQ = 2^−ΔΔCt^): being ΔCt the difference between the threshold cycle (Ct) of the target gene and the Ct of the housekeeping gene and ΔΔCt the difference between ΔCt of the sample and ΔCt of the calibrator. The calibrator corresponded to the sample transfected with miR-Neg.

### Cell growth assays

To assess the effect of *miR-550a-3p* on cell proliferation, cells were trypsinized at different intervals from transfection with miR-Neg or *miR-550a-3p*, and counted in a particle counter (Beckman Coulter Inc., Brea, California, USA). Results were expressed as percent variation in the number of *miR-550a-3p*-transfected cells compared with Neg-transfected cells.

The anchorage-independent growth assay was performed as described by Cuccuru et al. [21]. Briefly, 24 h after miR-Neg or *miR-550a-3p* transfection, cells were trypsinized and suspended in a medium containing 0.33% of agarose (Sigma–Aldrich, St. Louis, Missouri, USA) and was layered onto semisolid agarose (0.5% of agarose in medium) in duplicate on 9.6 cm^2^ dishes. After 10 days of incubation at 37 °C, cell colonies were stained with p-iodonitrotetrazolium violet (Sigma–Aldrich) and counted by Image J software.

### Western blot analysis

Whole-cell lysates were resolved by SDS-PAGE, transferred to nitrocellulose membranes, and probed with specific antibodies, as previously described [22]. The following primary antibodies were used: anti-Caspase-3 (#9662, 1:1000) and Caspase-9 (#9502, 1:1000) (Cell Signaling Inc, Beverly, MA, USA), anti-HSP90 alpha (TA332385, 1:2000; Origene, Rockville, MD, USA), anti-Cdc37 (sc-5617, 1:000), and anti-Raf-1 (sc-133, 1:1000) (Santa Cruz Biotechnology Inc, Santa Cruz, CA, USA), anti-Akt (610861, 1:1000; BD Biosciences, San Jose, CA, USA); anti-p53 (DO-7 M700101-2, 1:1000, Dako, Agilent, Santa Clara, CA, USA). Anti-Vinculin (V9131, 1:5000; Sigma–Aldrich) was used as an equal protein loading control. The filters were then incubated with the secondary peroxidase-linked whole antibodies and detailed in Material and Methods Supplementary Information. Bound antibody was detected using the Novex ECL, HRP Chemiluminescent substrate Reagent Kit (Thermo Fisher Scientific Inc). Membranes were cropped to allow simultaneous incubation of different primary antibodies on the same samples. Membranes were stripped and successfully reincubated with a second antibody, where appropriate. For the preparation of figures, we cropped the original western blot to generate the appropriate figure panels with the relevant lanes. The cropped image was then subjected to uniform image enhancement of contrast and brightness. Molecular weights were determined using the Precision Plus Protein™ Standard (Bio-Rad Laboratories, Hercules, California, USA), which yields a colorimetric image only and has been removed from the chemoluminescence blot image.

### Apoptosis assay

At different time points after transfection with miR-Neg or *miR-550a-3p*, floating and adherent cells were harvested and processed for apoptosis evaluation by TUNEL assay according to manufacturer’s instructions (Roche, Basel, Switzerland). The cells were subjected to FACS analysis (BD Accuri™ C6 Cytometer, Becton Dickinson, Basel, Switzerland).

### Cell invasion assay

Invasion assay was performed 72 h after transfection with miR-Neg or *miR-550a-3p* by using Transwell membranes previously coated with 3.47 μg Matrigel/well (Boyden chamber with 8 mm pore size filter in the inset chambers (Costar, Corning Inc., NY, USA)) according to the protocol of our previous studies [14]. Cells were suspended in 300 µL serum-free medium and seeded into the insert chambers. After 24 h of incubation at 37 °C in 5% CO_2_, cells that migrated into the bottom chamber containing 1 ml of complete medium were fixed in 95 % ethanol, stained with a solution of 0.4% sulforhodamine B in 0.1% acetic acid, photographed, and counted under an inverted microscope.

### In vivo experiments

Animal experiments were approved by the Ethics Committee for Animal Experimentation of INT, authorized by the Italian Ministry of Health according to the national law (Project approval code: 1120/2015-PR), and performed in compliance with international policies and guidelines. SCID mice (8-week-old female) were purchased from Charles River Laboratories (Charles River Laboratories, Wilmington, Massachusetts, USA). Cells were transfected with *miR-550a-3p* or miR-Neg for 24 h, and then subcutaneously injected into the mouse right flank (5 × 10^6^cells/mouse). Each experimental group was composed of five mice. Inoculated animals were inspected daily to establish the time of tumor onset. Tumor growth was measured every 2–3 days using a Vernier caliper. The subcutaneous tumor volume was calculated as follows: TV (mm^3^) = d^2^ × D / 2, where d and D are the shortest and the longest diameter, respectively. Volume inhibition percentage (TVI %) in tumors derived from *miR-550a-3p*- over Neg-transfected cells was calculated as follows: TVI% = 100 − (mean *miR-550a-3p* TV / mean Neg TV × 100).

### In silico prediction of miRNA targets

Putative targets of *miR-550a-3p* were selected using miRWalk2.0 (http://www.ma.uniheidelberg.de/apps/zmf/mirwalk) algorithm. Predicted targets of miRWalk2.0 are obtained by integration of predicted miRNA targets produced by 12 established miRNA-target prediction programs (miRWalk microt4, miRanda, mirbridge, miRDB, miRMap, miRNAMap, PicTar2, PITA, RNA22, RNAhybrid, and TargetScan). We only selected the targets predicted by at least six of these programs which employ different algorithm predictions.

### Gene expression profiling

Twenty-four hours after transfection with miRNA mimics (miR-Neg and *miR-550a-3p*) STO cells were lysed as described above. RNA was extracted from three independent transfections of STO cells with miRNA mimics and analyzed on Illumina BeadStudio v4 gene expression platform. Scanned images were collected using Illumina BeadStudio v3.3.8 and processed using the lumi package [23] from Bioconductor v3.0 [24]. Raw data were log_2_-transformed, normalized with Robust Spline Normalization, and filtered, keeping only probes with a detection *p*-value < 0.01 in at least one sample; probes not associated with the official gene symbol were removed. Expression data were deposited in the Gene Expression Omnibus repository (GEO) with accession number GSE165341. Differentially expressed genes between the two conditions were identified using the limma package [25], and significance was assessed by Benjamini–Hochberg false discovery rate (FDR) method in order to take into account the multiple-testing correction.

### Statistical analyses

The effect of *miR-550a-3p* ectopic expression in cell-based assays was assessed by using the nonparametric Wilcoxon or Kruskal–Wallis tests according to the number of considered groups [26] and corresponding *p*-values were estimated according to exact test or via Monte Carlo approaches. The nonparametric Sign Test was used to compare the sample distribution to a given value. For the animal experiment, a mixed model (with a compound symmetry covariance matrix) was fitted to assess the tumor growth (on a logarithmic scale) as a function of time and experimental groups (fixed factors) with mice considered as a random factor. For clinical data, relapse-free survival (RFS) was calculated as the time from surgery to the first relapse and overall survival (OS) as a time to death due to any cause. The prognostic role of *miR-550a-3p* expression (considered on its original continuous scale) on RFS and OS was investigated using a Cox regression model in a univariate fashion [27]. The relationship between *miR-550a-3p* expression and outcome was investigated using a regression model based on restricted cubic splines. Subsequently, *miR-550a-3p* was dichotomized according to the median value and the patterns of RFS and OS were estimated using the Kaplan–Meier method [28], and the survival curves were compared using log-rank tests. All statistical analyses were carried out with SAS (Statistical Analysis System, RRID:SCR_008567, version 9.4.; SAS Institute, Inc., Cary, NC) by adopting an alpha level of 5%.

## Results

### *miR-550a-3p* is downregulated and its expression is a prognostic factor in DMPM

**Fig. 1:**
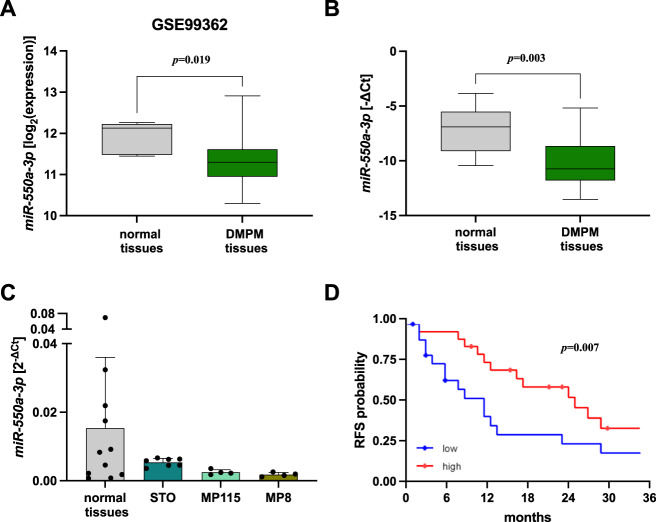
*miR-550a-3p* is downregulated and its expression is a prognostic factor in DMPM. **A** Boxplot depicting the *miR-550a-3p* endogenous expression levels assessed by miRNA expression profiling in DMPM (*n* = 51) and normal peritoneum (*n* = 5) specimens. Data were retrieved from GEO repository with accession number GSE99362, in the form of normalized data matrix [15]. Data are reported as log_2_-transformed normalized signal intensities for *miR-550a-3p* in each specimen. Each box indicates the 25th and 75th centiles of the distribution. The horizontal line inside the box indicates the median and the whiskers indicate the extreme measured values (Wilcoxon test, *p* = 0.019). **B** Boxplot depicting *miR-550a-3p* expression levels by quantitative real-time RT-PCR (qRT-PCR) analysis in normal tissues (*n* = 11) and primary tumor from DMPM patients (*n* = 45). Data are reported as −ΔCt. Each box indicates the 25th and 75th centiles of the distribution. The horizontal line inside the box indicates the median and the whiskers indicate the extreme measured values (Wilcoxon test, *p* = 0.003). **C** Dotplot depicting the expression levels of *miR-550a-3p* in DMPM cell lines (STO, MP8, and MP115 cells; *n* ≥ 4 independent experiments for each cell line) versus normal tissues (*n* = 11). Data were presented as the mean value of 2^−∆Ct^ ± SD. **D** Kaplan–Meier curves representing patient RFS according to *miR-550a-3p* expression, considered as a dichotomous variable; median *miR-550a-3p* levels were used as a threshold for the definition of *miR-550a-3p* categories (low and high) (log-rank tests, *p* = 0.007).

By comparatively assessing miRNA profiles in 51 DMPM and 5 normal mesothelium samples on a microarray platform (GSE99362) [15], we initially identified *miR-550a-3p* as a downregulated miRNA in tumors (*p* = 0.019) (Fig. 1A). The significantly reduced abundance of *miR-550a-3p* was then confirmed by qRT-PCR in an independent DMPM cohort, including 45 DMPM and 11 normal mesothelium samples (*p* = 0.003) (Fig. 1B). Consistently, *miR-550a-3p* showed a trend of down-modulation also in DMPM cell lines (STO, MP8, and MP115) developed in our lab from DMPM clinical samples (Fig. 1C). Thanks to the availability of clinico-pathogical and follow-up information of DMPM patients, we then investigated whether *miR-550a-3p* expression level is associated with patient clinical outcomes. Clinico-pathological characteristics of the cohort are reported in Table 1. Median follow-up was 31 months (interquartile, 11–136 months). Univariate Cox analysis revealed a statistically significant association between *miR-550a-3p* expression, considered on its continuous scale, and RFS (HR: 0.802, 95% CI: 0.671; 0.959, *p* = 0.015) (Supplementary Fig. 1). The significance was retained also when we pursued the analysis by dichotomizing the *miR-550a-3p* expression (*p* = 0.007) (Fig. 1D). No statistically significant association was observed between *miR-550a-3p* expression and patient OS (HR: 0.876, 95% CI: 0.711; 1.080), possibly because of the low number of events.

**Table 1:** Clinico-pathological characteristics of DMPM patients.

		No. patients (%)
Sex	Female	16 (36)
	Male	29 (64)
Lymph-node status^a^	Negative	33 (89)
	Positive	4 (11)
Subtype	Epithelioid	40 (89)
	Biphasic/sarcomatoid	4/1 (11)
Performance status	0	40 (89)
	1–2	5 (11)
Preoperative systemic chemotherapy	Yes	21 (47)
	No	24 (53)
Residual disease after CRS	No residual tumor	43 (96)
	Residual tumor ≤2.5 mm	2 (4)

^a^Data on N-stage were not available for eight cases.

### Ectopic expression of *miR-550a-3p* in DMPM cells counteracts growth and invasion and promotes apoptosis

**Fig. 2:**
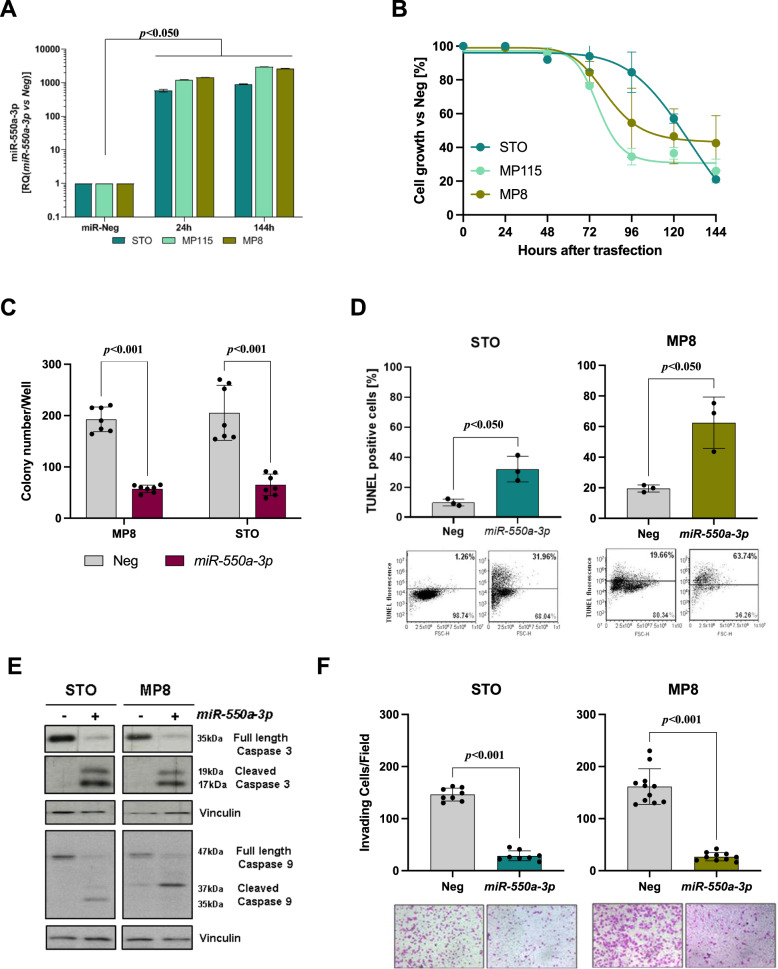
*miR-550a-3p* counteracts growth and invasion and promotes apoptosis in DMPM cells. **A** qRT-PCR of miRNA expression at different times after transfection (24 and 144 h) with respect to miR-Neg cells. Data were presented as RQ values. Means ± SD values are reported (Sign Test, *p* < 0.05 one-sided). **B** Antiproliferative effects of *miR-550a-3p* ectopic expression on DMPM cell lines. DMPM cell growth was assessed by cell counting after transient transfection with miRNA Negative controls (Neg) or *miR-550a-3p* at different times. Data are expressed as percentage cell proliferation of *miR-550a-3p*- versus Neg-transfected cells. Means ± SD values of three independent experiments are reported. **C** Effect of *miR-550a-3p* ectopic expression on anchorage-independent growth assay in STO and MP8 cells after 10 days. Data represented as mean colony number/well ± SD (Wilcoxon test, *p* < 0.001 one-sided). **D** Flow cytometric assessment of apoptosis by TUNEL assay. Transfected STO and MP8 cells (Neg and *miR-550a-3p*) were processed after 120 and 144 h, respectively. Data represented as mean percentage of TUNEL-positive cells ±SD of three independent experiments (Wilcoxon test, *p* < 0.05 one-sided). A representative dotplot of one experiment is reported below. **E** STO and MP8 cells were processed to assess cleavage of caspase-3 and 9 expression by western blot analysis at 120 and 144 h after miRNAs transfection, respectively. Vinculin was used as equal protein loading controls. Cropped images of selected proteins are shown. **F** Modified Boyden chamber with Matrigel-precoated membrane filter insert was used to measure in vitro invasiveness. Twenty-four hours after miRNA reconstitution, STO, and MP8 cell invasion was subsequently tested using a Matrigel-coated Transwell system. After 24 h of incubation, cells that migrated through the membrane were stained and representative fields were photographed. Original magnification, ×40. Invasion was quantified by counting cells in ≥8 random fields. Columns represent the average of the number of cells per field (Wilcoxon test, *p* < 0.001 one-sided). Representative pictures are reported below.

To assess the biological role of *miR-550a-3p* in DMPM, we transiently transfected the three cell lines with synthetic *miR-550a-3p* mimic and miRNA negative control. A marked increase in miRNA abundance was observed in all cell lines at 24 h from transfection with the miRNA mimic, which was still appreciable, to a comparable extent, at 144 h (Fig. 2A, *p* < 0.05). *miR-550a-3p* ectopic expression inhibited the proliferation of all DMPM cell lines in a time-dependent manner, although a more rapid cell growth decline was observed in MP8 and MP115 cells than in STO cells (Fig. 2B). Moreover, results obtained in an anchorage-independent growth assay showed the ability of *miR-550a-3p* to significantly reduce the clonogenic potential of STO and MP8 cells (*p* < 0.001) (Fig. 2C). The antiproliferative effects of *miR-550a-3p* were paralleled by the induction of a marked apoptotic response in both cell lines, as indicated by the enhanced percentage of TUNEL-positive cells compared to cells transfected with the miRNA negative control (*p* < 0.05) (Fig. 2D), as well as by the presence of the cleaved forms of caspase-3 and caspase-9 (Fig. 2E). Interestingly, *miR-550a-3p* was also able to significantly reduce the invasive capabilities of DMPM cells in a matrigel-based assay (p < 0.001) (Fig. 2F). The proapoptotic effects of *miR-550a-3p* reconstitution were observed also in MP115 cells (Supplementary Fig. S2).

**Fig. 3:**
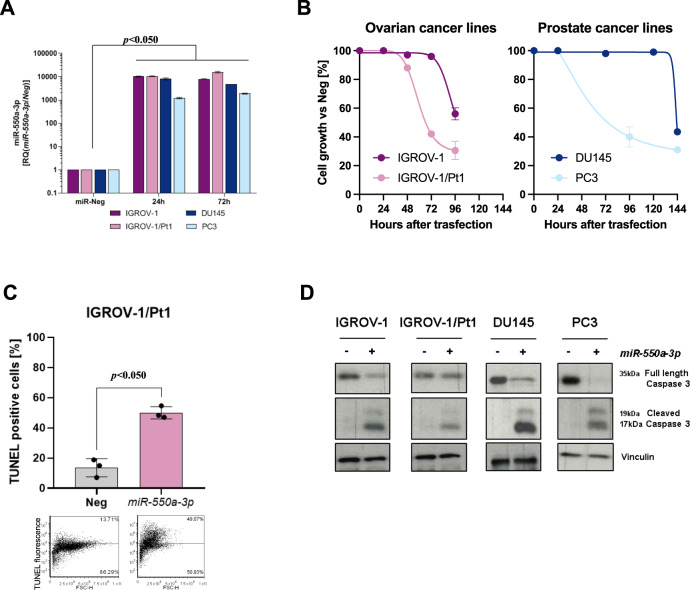
*miR-550a-3p* induces antiproliferative and proapototic effects in ovarian and prostate cancer cells. **A** Quantitative real-time RT-PCR of *miR-550a-3p* expression. Data were presented as RQ values with respect to miR-Neg at different times after transfection (24 and 72 h). Means ± SD values are reported (Sign Test, *p* < 0.05 one-sided). **B** Antiproliferative effect of *miR-550a-3p* at different time upon transfection. Cells were transfected with either miR-Neg control or *miR-550a-3p* and counted by cell counter at different time after transfection. Data are expressed as percentage cell proliferation of *miR-550a-3p*- versus miR-Neg-transfected cells (100%). Means ± SD values of three independent experiments are reported. **C** Flow cytometric assessment of apoptosis by TUNEL assay at 72 h after transfection in IGROV-1/Pt1 cells. Data represented as mean ± SD of three independent experiments (Wilcoxon test, *p* < 0.05 one-sided). A representative dotplot of one experiment is reported below. **D** Analysis of caspase-3 expression at 72 h after *miR-550a-3p* reconstitution in ovarian and prostate cancer cells. Vinculin was used as equal protein loading controls. Cropped images of selected proteins are shown.

To assess whether the tumor-suppressive functions of *miR-550a-3p* could be extended to models of tumor types other than DMPM, we ectopically expressed *miR-550a-3p* in two ovarian cancer (IGROV-1 and its platinum-resistant derivative IGROV-1/Pt1) and two prostate cancer (DU145 and PC3) cell lines (Fig. 3A, *p* < 0.05). *miR-550a-3p* overexpression was found to inhibit cell growth in all cell lines, although to a variable extent and with different kinetics (Fig. 3B), and to induce an apoptotic response, as detected by the increase in the percentage of TUNEL-positive cells (*p* < 0.05) (Fig. 3C and Supplementary Fig. S2) and/or the presence of the cleaved form of caspase-3 (Fig. 3D).

### *miR-550a-3p* impairs DMPM growth in a xenograft model

**Fig. 4:**
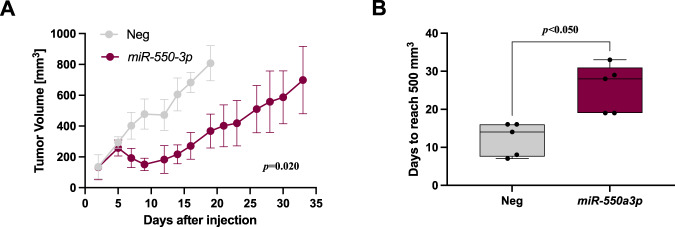
*miR-550a-3p* impairs DMPM growth in a xenograft model. STO cells transiently transfected with *miR-550a-3p* or miR-Neg vector (5 × 10^6^) for 24 h were immediately implanted subcutaneously into the right flank of nude mice. **A** Tumor growth volume (mm^3^) was measured with a Vernier caliper on indicated days after cell injection. Dots indicated the mean values and the whiskers indicate the 95% confidence intervals (Mixed model, *p* = 0.02 one-sided). **B** Comparison between the times to reach the size of 500 mm^3^ for tumors obtained from STO cells trasfected with miR-Neg or *miR-550a-3p*. The horizontal line inside the box indicates the median and the whiskers indicate the extreme values (Wilcoxon test, *p* < 0.050).

The in vitro tumor growth inhibitory effect of *miR-550a-3p* was challenged in the in vivo setting by subcutaneously transplanting STO cells transiently transfected with the miRNA mimic and the negative control into nude mice to generate xenografts. No differences were appreciable in the tumor take rate, which was 100% in both experimental groups. However, the growth of tumors originating from *miR-550a-3p* overexpressing cells was significantly delayed compared to those arising from control cells throughout the experiment (*p* = 0.02) (Fig. 4A, B), with a maximum tumor volume inhibition of 59% recorded at 19 days after cell inoculum. In light of the in vitro results, such a growth delay might be ascribed to reduced cell proliferation (Fig. 2B, C) and limited local invasive capabilities (Fig. 2F) of *miR-550a-3p* overexpressing cells.

### *HSP90AA1* is a functional target of *miR-550a-3p*

**Fig. 5:**
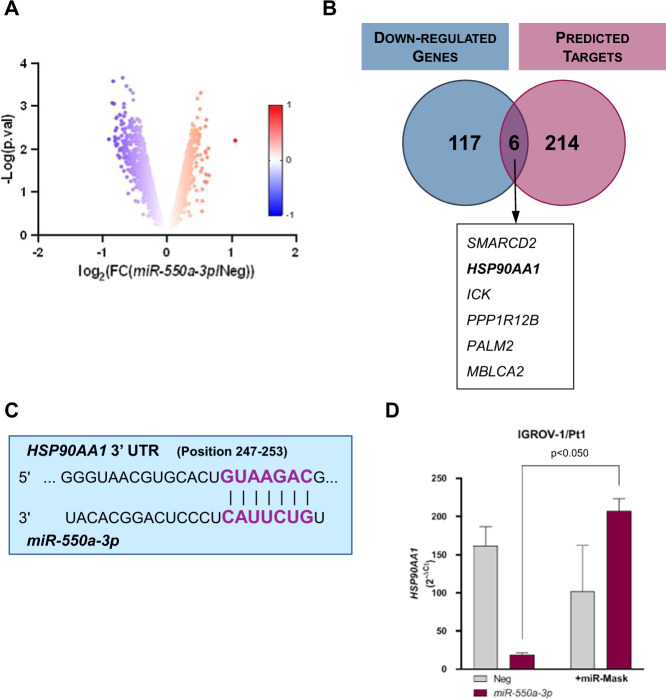
*HSP90AA1* is a functional target of *miR-550a-3p*. **A** Volcano plot of genes differentially expressed in *miR-550a-3p-*transfected STO cells compared to miR-Neg cells, as measured by microarray analysis; the red–blue color map is used as a graphical visualization of the magnitude of log_2_ fold change (FC) representing the positive and negative values, respectively. **B** Venn diagram of predicted *miR-550a-3p* targets according to at least 6 different algorithms, and significantly downregulated genes upon *miR-550a-3p* ectopic expression. In the box, the list of common elements between genes identified by microarray analysis and predicted *miR-550a-3p* targets is reported. **C** Representation of *miR-550a-3p* duplexed with the 3′UTR of *HSP90AA1* mRNA, as from TargenScanHuman (http://www.targetscan.org/vert_72). **D** qRT-PCR showing *HSP90AA1* mRNA expression levels in IGROV-1/Pt1 cells transfected with *miR-550a-3p* mimic, in the presence or absence of miR-Mask, compared to control cells, normalized to *GAPDH*. Data are reported as relative quantity (2^−ΔCt^) ± SD with respect to Neg cells (Wilcoxon test, *p* < 0.050 one-sided).

In the search for molecular determinants through which *miR-550a-3p* affects proliferation, apoptosis, and invasion, we conducted an in silico target prediction analysis by using the miRWalk2.0 tool [29]. The 220 predicted targets were then crossed with the 123 genes found to be downregulated in STO cells following *miR-550a-3p* ectopic expression, which we identified by comparatively evaluating gene expression profiles of *miR-550a-3p* overexpressing and negative control cells (GSE165341) (Fig. 5A and Supplementary Table 1). The resulting intersection included six genes, which are listed in Fig. 5B. Among them, we focused on the *heat shock protein 90 alpha family class A member 1* (*HSP90AA1*) gene coding for the heat shock protein 90 alpha (Hsp90α), the stress-inducible isoform of the molecular chaperone Hsp90, which interacts and supports numerous proteins that promote oncogenesis and is associated with each hallmark of cancer [30]. One 7mer-m8 site (intended as having an exact match to positions 2–8 of the mature miRNA, including the seed and position 8) complementary to *miR-550a-3p* is actually evident in position from 247 to 253 of *HSP90AA1* 3’UTR (Fig. 5C). To functionally address this point, a target protection approach was pursued. Specifically, IGROV-1/Pt1 cells overexpressing *miR-550a-3p* were transfected with a miR-Mask, a custom oligonucleotide designed to be fully complementary to *miR-550a-3p* binding site within *HSP90AA1* 3’UTR, to assess whether the disruption of miRNA-target interaction could abolish the *miR-550a-3p*-mediated repression of *HSP90AA1* mRNA. Notably, the miR-Mask was able to completely restore *HSP90AA1* transcript levels, thus confirming *HSP90AA1* as a direct target of the miRNA (Fig. 5D).

**Fig. 6:**
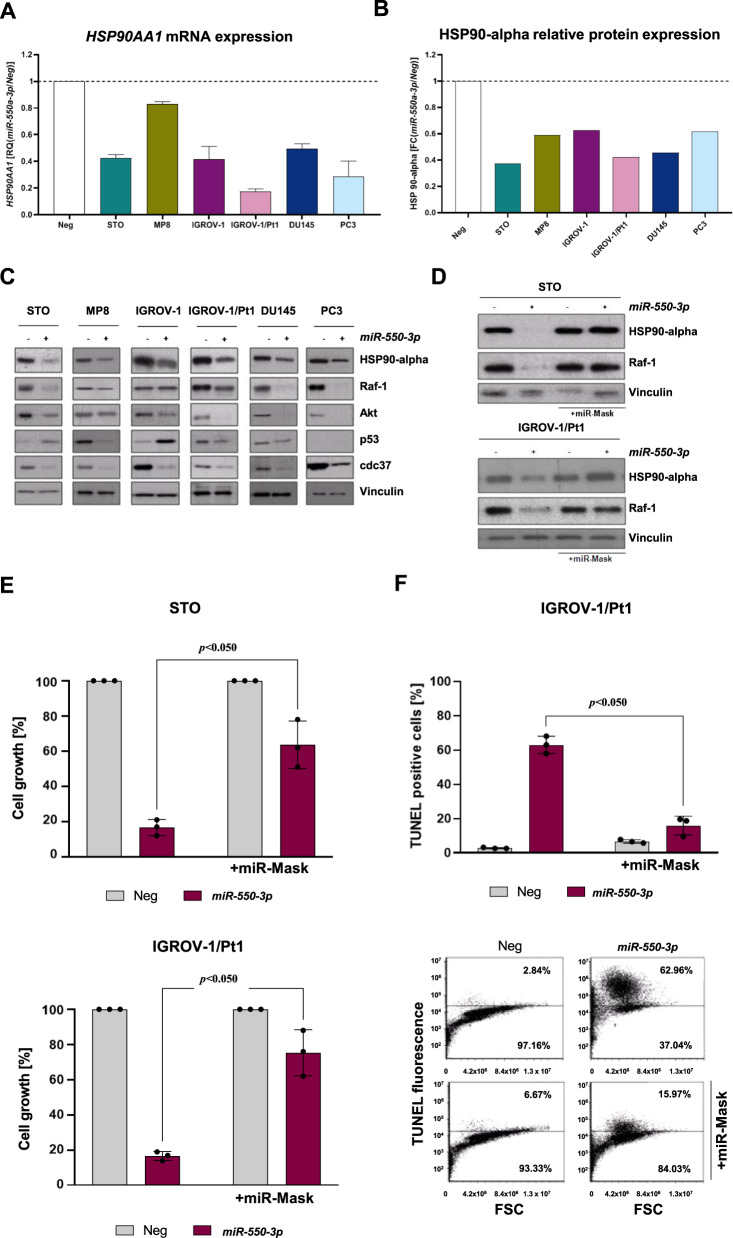
*HSP90AA1* suppression is a determinant of *miR-550a-3p*-mediated antiproliferative and proapoptotic effects. **A** qRT-PCR showing *HSP90AA1* mRNA amount in *miR-550a-3p*-transfected tumor cells normalized to *GAPDH*. Data are reported as relative quantity (RQ) with respect to miR-Neg cells. Dotted line represents miR-Neg-transfected cells value. Means ± SD values are reported (Sign Test, *p* < 0.05 one-sided). **B** Relative quantification of HSP90 alpha protein levels, in *miR-550a-3p*-transfected cells compared to respective miR-Neg transfectants, as detected by western blot reported in **C**. Data are reported as fold change (FC) with respect to miR-Neg cells. Dotted line represents miR-Neg-transfected cells value. **C** Analysis of HSP90 alpha, its client (Raf-1, Akt, p53) and cochaperone Cdc37 expression in tumor cells upon transfection with miR-Neg or *miR-550a-3p* by western blot analysis. Vinculin was used as equal protein loading controls. Cropped images of selected proteins are shown. **D–F** IGROV-1/Pt1 and STO cells were transfected with miRNA (miR-Neg or *miR-550a-3p*) in the presence or absence of miR-Mask and processed 96 h after transfection. **D** Western blot analysis showing HSP90 alpha and Raf-1 protein expression upon *miR-550a-3p* reconstitution in the presence or absence of miR-Mask. Vinculin was used as equal protein loading controls. Cropped images of selected proteins are shown. **E** Antiproliferative effects of *miR-550a-3p* ectopic expression in the presence or absence of miR-Mask. Data are expressed as percentage cell proliferation of *miR-550a-3p*-transfected cells referred to the negative control miRNA (100%). Means ± SD values of three independent experiments are reported (Wilcoxon test, *p* < 0.05 one-sided). **F** Flow cytometric assessment of apoptosis by TUNEL assay in IGROV-1/Pt1 cells at 96 h after *miR-550a-3p* transfection in the presence or absence of miR-Mask. Data represented as mean ± SD of three independent experiments (Wilcoxon test, *p* < 0.05 one-sided). A representative dotplot of one experiment is reported.

Ectopic expression of *miR550a-3p* in DMPM, ovarian cancer, and prostate cancer cell lines consistently decreased *HSP90AA1* expression at both mRNA and protein levels (Fig. 6A,B). In addition, *miR-550a-3p* overexpression caused a decline in the abundance of HSP90 client proteins (Raf-1, Akt) and co-chaperons (Cdc37) (Fig. 6B). Interestingly, consistent with what was observed with HSP90 inhibitors, such as 17-(allylamino)-17-demethoxygeldanamycin [31, 32], *miR-550a-3p* induced opposing effects on wild-type and mutant p53. Specifically, p53 was upregulated in wild-type p53-expressing cell lines (STO and IGROV-1) and downregulated in mutant p53 tumor cells (MP8, IGROV-1/Pt1, and DU145) (Fig. 6C).

Finally, to prove that the oncosuppressive effects induced by *miR-550a-3p* are mediated by direct targeting of *HSP90AA1*, STO, and IGROV-1/Pt1 cells were co-transfected with the *miR-550a-3p* mimic and the miR-Mask. The presence of miR-Mask was able to almost completely restore HSP90 alpha protein expression levels (Fig. 6D), and also partially prevented Raf-1 downregulation by *miR-550a-3p* in both cell lines (Fig. 6D). Moreover, the rescue of HSP90 alpha protein expression by the miRNA-Mask reduced the antiproliferative (*p* < 0.05) (Fig. 6E) and proapoptotic effects (*p* < 0.05) (Fig. 6F) induced by *miR-550a-3p*.

Collectively, these results suggest that *miR-550a-3p* directly targets *HSP90AA1* and that the observed tumor-suppressive effects caused by miRNA ectopic expression are mediated, at least in part, by the interference with the HSP90 alpha-client protein axis.

## Discussion

Compelling evidence about the functional involvement of miRNAs in cancer onset and progression has emerged from a huge amount of studies carried out on experimental models and clinical samples of a variety of human tumor types. However, concerning malignant mesothelioma, almost all available information has been generated on the pleural variant, and only a couple of studies dealt with DMPM [33]. Specifically, El Bezawy et al. [14] showed that *miR-34a* is downregulated in DMPM clinical specimens and cell lines. Moreover, *miR-34a* reconstitution in DMPM cells was found to inhibit proliferation and tumorigenicity, to induce an apoptotic response, and to decline invasion ability, mainly through the downregulation of its targets c-MET and AXL and the interference with the activation of downstream signaling. Cimino-Reale et al. [15] showed that the ectopic expression of *miR-380-5p*, a miRNA negligibly expressed in telomerase-positive DMPM clinical specimens, negatively interferes with telomerase activity and growth of DMPM cell lines by targeting the telomerase associated protein 1.

In this study, we demonstrated that *miR-550a-3p* is downregulated in DMPM clinical samples and cell lines, and that its expression levels are inversely associated with patients’ outcomes, thus suggesting a possible oncosuppressive role of the miRNA in the disease. Consistently, functional experiments revealed that *miR-550a-3p* ectopic expression in DMPM cells impairs proliferation and invasiveness, enhances apoptosis, and reduces the growth of xenografts in mice. Interestingly, we also observed antiproliferative and proapoptotic effects following *miR-550a-3p* ectopic expression in prostate cancer and ovarian cancer cell lines, thus suggesting that the miRNA is endowed with tumor-suppressive functions also in these tumor types.

By crossing in silico predicted targets and genes found to be downregulated in DMPM cells following *miR-550a-3p* ectopic expression, we identified and focused on *HSP90AA1* as a direct target through which the miRNA exerts, at least in part, its oncosuppressive functions. Indeed, *HSP90AA1* is the gene coding for HSP90 alpha, the stress-inducible isoform of HSP90 belonging to the family of molecular chaperones that have a key role in the stabilization of oncogenic proteins, such as Raf-1, ErbB2, Akt, and mutant p53, thus promoting survival of cancer cells [34]. The involvement of HSP90 in all of the hallmarks of cancer supports the functional role of HSP90 alpha in the pleiotropic effects induced by *miR-550a-3p* reconstitution in tumor cells. Chaperone signaling pathways are dysregulated in a wide range of tumors [35]. Recent studies reported that *HSP90AA1* expression levels are upregulated in tissue and plasma of several cancers and correlated with poor prognosis [36–38]. As a consequence of *HSP90AA1* inhibition, the reconstitution of *miR-550a-3p* resulted in the degradation of HSP90 client proteins, including Raf-1 and Akt. The involvement of HSP90 was also supported by a different p53 modulation upon *miR-550a-3p* reconstitution in our cellular models. Specifically, p53 was upregulated in wild-type p53-expressing cell lines (STO and IGROV-1 cells) and downregulated in mutant p53 tumor cells (MP8, IGROV-1/Pt1, and DU145 cells). These p53 status-dependent modulations have been previously described after treatment of cancer cells with HSP90 inhibitors, i.e., geldanamycin [31, 32], and are consistent with the cytotoxic and proapoptotic effects we observed in all our cell models. Akt and Raf-1 kinases play an important role in the control of pathways that regulate proliferation and apoptosis. The inhibition of functional HSP90 could coordinately block the transduction of growth factor signaling via the Akt and Raf-1 pathways. Indeed, Jones et al. previously reported that the depletion of Akt and Raf-1 tyrosine kinases after HSP90 inhibition resulted in apoptotic cell death as a consequence of the loss of prosurvival signals [39]. Moreover, the depletion of cdc37, an intracellular cofactor of HSP90, following *miR-550a-3p* reconstitution may further contribute to the disruption of the HSP90 chaperone machinery by impairing the association of client proteins and preventing protein maturation. Such blockage has been previously described to suppress multiple pathways and to induce growth inhibition in human cancer cells [40, 41]. Consistent with an oncosuppressive role of *miR-550a-3p*, Ho et al. [38] previously reported that the miRNA was downregulated in breast cancer clinical samples and cell lines, and that its ectopic expression in breast cancer cells impaired proliferation, invasion, and migration as well as tumorigenesis in a xenograft mouse model. Conversely, Yang et al. [42] showed that the *miR-550a-3p* was upregulated in non-small cell lung cancer (NSCLC) tissues compared to surrounding normal tissues, and that forced overexpression of the miRNA in NSCLC cells promoted proliferation, invasion, and migration through the inhibition of TIMP2. Xiong et al. [43] reported that *miR-550a-3p* was one of the most upregulated miRNAs in plasma extracellular vesicles from melanoma patients compared to healthy individuals, and that a signature consisting of high expression levels of *miR-550a-3p*, CDK2, and POLR2A and low expression levels of *miR-150-5p* was associated with reduced overall survival of melanoma patients. Again, by analyzing TCGA miRNA expression profiles of hepatocellular carcinoma, Qin et al. [44, 45] showed that high *miR-550-3p* expression levels were associated with reduced progression-free survival.

The finding that *miR-550a-3p* displays opposite roles in different human tumor types is not surprising since miRNAs have been acknowledged to exert either tumor-suppressive or oncogenic functions depending on their expression levels, cell/tissue context, and availability of target genes [12]. This notion reinforces the necessity of a detailed understanding of the functions exerted by a specific miRNA and the precise identification of its key targets relevant to the disease of interest in view of its possible exploitation as a novel target/tool for anticancer therapy.

In conclusion, due to the inherent resistance of DMPM to chemotherapy and the lack of alternative effective treatments for patients who are not suitable for or fail after CRS + HIPEC, novel miRNA-based therapeutic approaches are highly desirable. Preclinical data generated in the present study form a solid foundation for promoting the clinical use of a *miR-550a-3p*-based approach as a novel therapeutic strategy to be pursued for DMPM and potentially extended to other more frequent tumor types, such as prostate and ovarian cancers.

miRNA mimics and inhibitors already entered the clinical therapeutic armamentarium in oncology. However, the number of clinical studies with miRNA modulators carried out so far is still limited. The first miRNA-based compound entering a phase I study in patients with advanced solid tumors was MRX34, a *miR-34a* mimic encapsulated in lipid nanoparticles, showing evidence of activity in a subset of patients. However, the study was closed early due to serious immune-mediated adverse events [46]. The activity of a *miR-16* mimic delivered by bacterial minicells targeted to EGFR (TargomiRs) was then evaluated in a phase 1 study of patients with recurrent malignant pleural mesothelioma, showing a partial response in one out of 22 evaluable patients [47, 48]. Moreover, a locked nucleic acid-modified oligonucleotide inhibitor of *miR-155* (Cobomarsen, MRG-106) is currently under clinical investigation in patients with cutaneous T-cell lymphoma [49].

A major constraint towards a concrete application of miRNA-based strategies to human cancer therapy is related to the need for safe and efficient delivery. Although several approaches have been tested, including lipid carriers, oligonucleotides with different chemical modifications, viral vectors, and, more recently, extracellular vesicles, strong in vivo evidence for improved efficiency and limited toxicity is still lacking. Dosage concerns and off-target effects also remain major challenges to be overcome for the clinical development of miRNA-based therapies as well as the need for an improvement of currently available information concerning pharmacokinetics of miRNA mimics and inhibitors [50]. Moreover, it must be considered that, as anticancer drugs, also miRNA-based therapeutics might activate resistance mechanisms. In this context, El Bezawy et al. [14] provided evidence of a cytoprotective mechanism against *miR-34a* reconstitution in DMPM cells through the persistent activation of ERK1/2 and Akt signaling.

## Supplementary information

Material and Methods Supplementary Information

Legends supplementary information

Supplementary figureS1

Supplementary figureS2

Table S1 Supplementary Information

## Data Availability

All data generated and/or analyzed during this study are included in this article and its supplementary information files.
