# Supplementary material for: miR-550a-3p is a prognostic biomarker and exerts tumor-suppressive functions by targeting HSP90AA1 in diffuse malignant peritoneal mesothelioma
Source: Cancer Gene Ther. 2022 Mar 29;29(10):1394–404. doi: 10.1038/s41417-022-00460-7 (PMC9576593; doi:10.1038/s41417-022-00460-7)
Supplement: Supplementary file 1 — Material and Methods Supplementary Information [file 41417_2022_460_MOESM1_ESM.docx]

**Material and Methods Supplementary Information**

***Western-blot analysis***

The filters were incubated with the secondary peroxidase linked whole antibodies:

anti- Caspase 3 1:2000; anti-Caspase 9 1:2000, anti-HSP90 alpha 1:5000; anti-Cdc37 1:5000, anti-Raf-1 1:5000, anti-Akt 1:2000; anti-p53 1:2000 for p53 wild-type cells, 1:5000 for p53 mutated cell lines; anti-Vinculin 1:8000

***RNA retrotranscription***

Reverse-transcription reaction components of miScript SYBR Green PCR Kit (Qiagen)

Component Volume/reaction

5x miScript HiSpec Buffer 4 µl

10x miScript Nucleics Mix 2 µl

miScript Reverse Transcriptase Mix 2 µl

Template RNA 1 µg (volume total 10 µl)

RNase-free water Variable

Total volume 20 µl

Reverse-transcription master mix with template RNA was:

- Incubate for 60 minutes at 37ºC.

- Incubate for 5 minutes at 95ºC to inactivate miScript Reverse Transcriptase Mix and

place on ice.

cDNA was diluted in RNase-free water and proceed with real-time PCR immediately.

***miScript SYBR Green PCR Kit for expression analysis of miRNAS (miR-550a-3p and SNORD48)***

PCR Reaction mix:

**Component per well (96 well)**

2x QuantiTect SYBR Green PCR Master Mix 12.5 µl

10x miScript Universal Primer 2.5 µl

Specific Primer 2.5 µl

RNase-free water 5 µl

Template cDNA 2.5 µl

Total volume 25 µl

The reaction was carried out in a 96-well PCR plate at 95 °C for 15 min followed by 40 cycles of 94 °C for 15 sec, 55 °C for 30 sec and 70 °C for 30 sec, followed by dissociation step to distinguish specific from non-specific amplification products. Each sample was analyzed in triplicate. Amplifications were run on the 7900HT Fast Real-Time PCR System (Applied Biosystem).

***TaqMan®gene expression assays for detection of HSP90AA1 and GAPDH mRNA***

PCR Reaction mix:

**Component per well (96 well)**

TaqMan®gene Fast Universal PCR Master Mix 2x 5 µl

Specific Primer 0.5 µl

RNase-free water 2 µl

Template cDNA 2.5 µl

Total volume 10 µl

The reaction was carried out in a 96-well PCR plate at 95 °C for 20 min followed by 40 cycles of 95 °C for 1 sec, 60 °C for 20 sec. Each sample was analyzed in triplicate.
