# Supplementary material for: miR-550a-3p is a prognostic biomarker and exerts tumor-suppressive functions by targeting HSP90AA1 in diffuse malignant peritoneal mesothelioma
Source: Cancer Gene Ther. 2022 Mar 29;29(10):1394–404. doi: 10.1038/s41417-022-00460-7 (PMC9576593; doi:10.1038/s41417-022-00460-7)
Supplement: Supplementary file 2 — Legends supplementary information [file 41417_2022_460_MOESM2_ESM.docx]

**Supplementary information**

**Supplementary Fig. 1**. Relapse-free survival (RFS) curve for *miR-550a-3p*.The curve depicts the predicted RFS probability according to the *miR-550a-3p* expression levels considered on its continuous scale.

**Supplementary Fig. 2.** Cleavage of caspase 3 (A) and TUNEL assay (B) were performed in *miR-550a-3p* over-expressing MP115 and IGROV-1 cells 144 and 72 hours after transfection, respectively.

**Supplementary Table S1.** List of putative *miR-550a-3p* gene targets by miRNA target bioinformatic prediction method miRWalk 2.0 and of differentially expressed genes that were identified by microarray analysis performed in *miR-550a-3p* over-expressing STO cells.
