# Supplementary figures and images for: miR-550a-3p is a prognostic biomarker and exerts tumor-suppressive functions by targeting HSP90AA1 in diffuse malignant peritoneal mesothelioma
Source: Cancer Gene Ther. 2022 Mar 29;29(10):1394–404. doi: 10.1038/s41417-022-00460-7 (PMC9576593; doi:10.1038/s41417-022-00460-7)

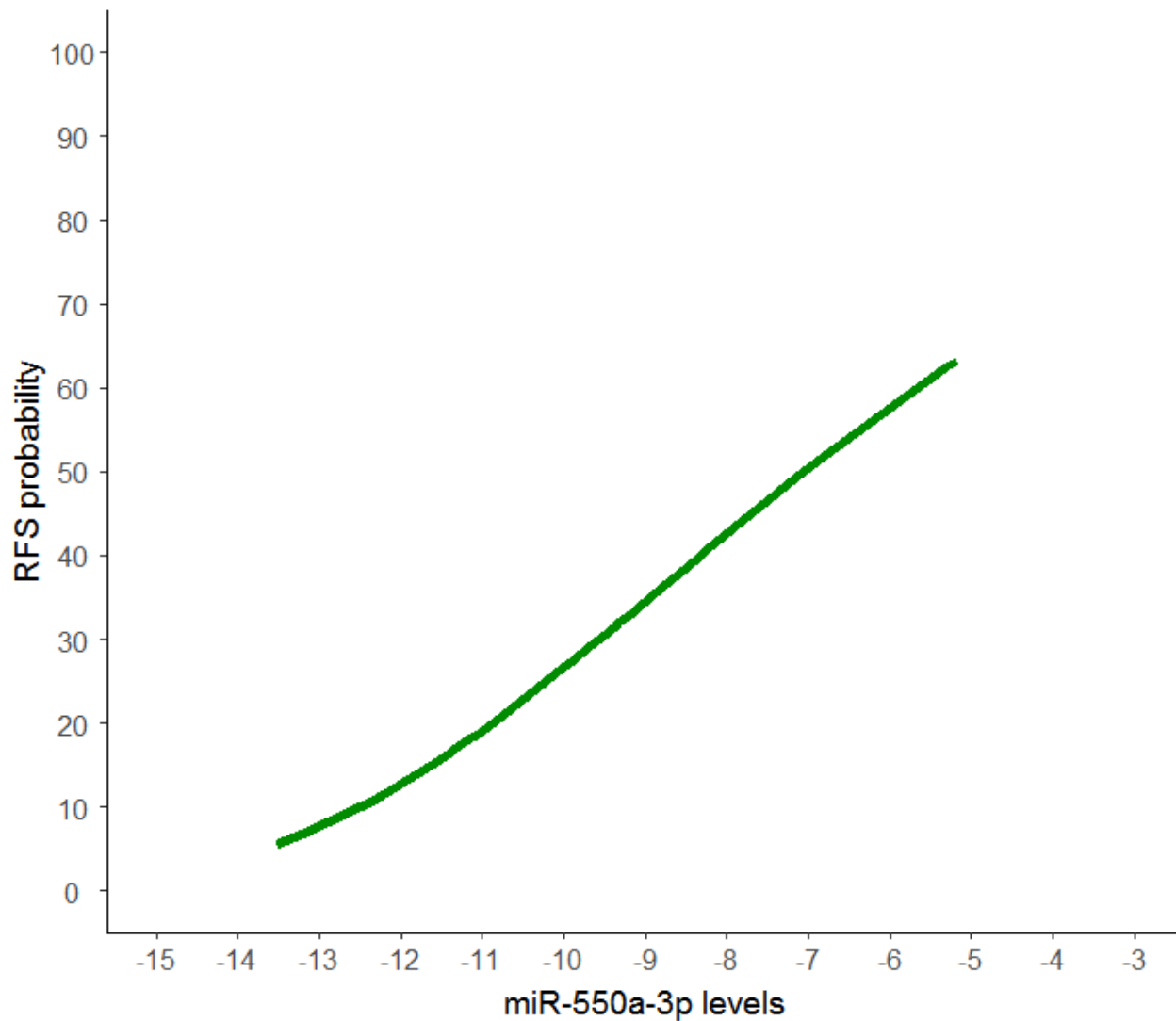

**Supplementary Figure 1**

Supplement: Supplementary file 3 — Supplementary figureS1 [file 41417_2022_460_MOESM3_ESM.pdf]

**A**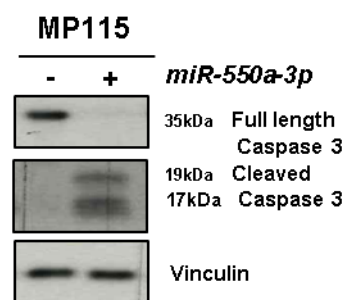**B**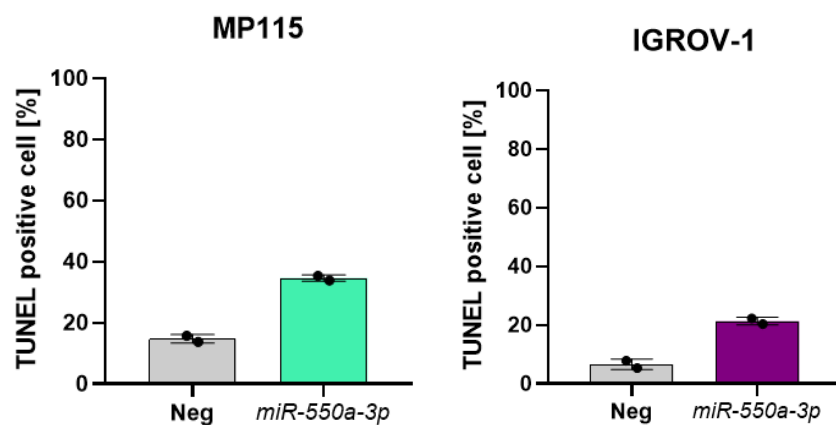**Supplementary Figure 2**

Supplement: Supplementary file 4 — Supplementary figureS2 [file 41417_2022_460_MOESM4_ESM.pdf]
